# Reflections on the first 8 years of *Epilepsia Open*

**DOI:** 10.1002/epi4.13010

**Published:** 2024-07-31

**Authors:** Aristea S. Galanopoulou

**Affiliations:** ^1^ Saul R. Korey Department of Neurology Albert Einstein College of Medicine Bronx New York USA; ^2^ Dominick P. Purpura Department of Neuroscience Albert Einstein College of Medicine Bronx New York USA; ^3^ Isabelle Rapin Child Neurology Division Albert Einstein College of Medicine Bronx New York USA

On September 1, 2024, I will be completing my 8‐year term as Editor‐in‐Chief of *Epilepsia Open* and will be handing over its leadership to the new Editor‐in‐Chief Merab Kokaia. It has been a privilege serving at its helms since 2016, the year when *Epilepsia Open* was created, alongside Dieter Schmidt, Gary Mathern, and Xuefeng Wang initially and Dong Zhou since 2018, as well as an esteemed group of associate editors who were instrumental in establishing this newly formed open access journal of the ILAE, *Epilepsia Open*, as one of the top epilepsy journals.

*Epilepsia Open* was created in 2016 to absorb the meritorious epilepsy research manuscripts that would not be published in the existing ILAE journals as well as provide a fully open access publishing platform responding to the growing calls for transparency and public access of funded research.^1^ In parallel, in epilepsy, there was an international movement to improve infrastructure and research practices so as to improve translation of preclinical research findings. *Epilepsia Open* became a suitable publishing platform to accomplish this, implementing policies that promoted transparency and rigorous scientific reporting, and minimized publishing bias by considering negative, confirmatory studies, and preliminary reports,^2^ as long as they have scientific and translational merit and importance. The journal also became a common platform for publishing International League Against Epilepsy (ILAE) reports particularly the reports of the ILAE/American Epilepsy Society (AES) Joint Translational Task Force, such as on preclinical models and rodent electrophysiological studies.^3^, ^4^, ^5^, ^6^, ^7^, ^8^, ^9^, ^10^, ^11^, ^12^ Importantly, *Epilepsia Open* attracted articles on global health as well as reports of research collaboratives and consumer‐led funding organizations reporting on research initiatives and progress or consensus statements on topics relevant to specialized areas of epilepsy research.

We further instituted article formats that would enable multicenter collaborative studies to publish their design and data as a package of single‐site short article reports and an overall across‐sites synthesis of findings, providing more insights into site‐specific versus across‐site findings as well as opportunities for crediting the work of key collaborators who would otherwise be diluted among multi‐author publications.^13^

Submissions to *Epilepsia Open* transformed through the years to a more author‐friendly free format, to make it easier for the authors to obtain feedback on whether their work is publishable in our journal, before committing to our preferred manuscript format. Realizing that this is an ILAE journal with a responsibility to address the diversity in sociopolitical, language, cultural, and training background of the authors, our editors have been addressing such issues with sensitivity often working with the authors to achieve a quality level that is suitable for our journal.

In 2018, we initiated the Epilepsia Open annual prizes to reward the best basic science and clinical original research published in the journal the year prior by young investigators. Being a fully open‐access journal carries a heavy responsibility in improving the clarity of published content, especially in a field where specialized terms and expressions may challenge the understanding of research advances by nonspecialists and the broader community, which looks upon scientific publications for the promise of new therapies, tools, and scientific insights that could transform the lives of those affected with seizures or epilepsies. To address this, a lived experience editorial position was initiated to advise and work with us in making the journal more relevant in these aspects. Some of the format changes have already been instituted, such as the plain summary, graphical abstract, and our inaugural lived experience editor, Action Amos, has been instrumental in providing input and developing new strategies to serve this goal.

In an effort to bring the editorial and peer review process closer to the young generation of investigators, we initiated the editorial internships, commencing in 2023 with our four inaugural editorial interns Pedro Viana, Naoto Kuroda, Servando Jesus Medel‐Matus, and Giulia Sofia Cereda. Our interns have been engaged in peer review of submitted articles, a curriculum of “meet the editors” sessions that exposed them to various styles of editorial handling of manuscripts, depending on the research area, and are currently guest editing a special issue on neurotechnology. The call for submissions on this special issue's topic is at: https://onlinelibrary.wiley.com/pb‐assets/assets/24709239/Neurotechnology%20in%20Epilepsy‐1716488051.pdf.

During these first 8 years of the journal, there has been a dramatic growth in terms of the usual metrics that reveal the success of a journal. Based on the remarkable progress of the journal, *Epilepsia Open* received expedited admission to Science Citation Index Expanded (SCI‐E) and became fully indexed in 2021, receiving an outstanding first impact factor of 4.026 (2022). The popularity of the journal has grown, marked by a tripling of original submissions and almost doubling of published articles and full‐text article views over the last year. To meet this growing research output, in 2024, *Epilepsia Open* increased the number of annual regular issues from 4 to 6. The pool of authors that submit to and publish at our journal has increasingly become more diverse, attracting submissions from 56 countries in 2023, compared to 27 in 2018. Despite a mild drop in the impact factor to 2.8, a result of post‐COVID‐19 submissions and of the significantly increased research output of the journal, the journal steadily holds its high rank as a top tier epilepsy journal, and has improved its standing among journals of the same area, being at Q2 among clinical neurology and Q2 among neuroscience journals, based on the Clarivate databases.

I am grateful to the ILAE for this precious opportunity to be involved in the development of this new open access journal, the *Epilepsia Open* editorial board members for their diligent work in establishing this journal as a competitor and top‐tier epilepsy journal, and the publishing support of Wiley staff who worked with us closely over the last 8 years. In particular, I am thankful for the support and thoughtful advice of Dieter Schmidt and Dong Zhou with whom I have had the privilege of working with at the journal's leadership positions the longest. I would be remiss had I not mentioned Laurie Beninsig, our managing editor, who tirelessly attends to every change, question, or initiative we entertain, lends her experience to every discussion we have on the future of the journal, and is the portal between authors and editors. Most importantly, I would like to thank the authors who trusted us with their work, helping us shape the identity of the journal. I am looking forward to observing the journal's continuing success during the next period in the journal's development, under the leadership of Merab Kokaia.

## CONFLICT OF INTEREST STATEMENT

I confirm that I have read the Journal's position on issues involved in ethical publication and affirm that this report is consistent with those guidelines. AS Galanopoulou is the Editor‐in‐Chief of Epilepsia Open, associate editor of Neurobiology of Disease, and receives royalties from Elsevier and Medlink for publications.

## Data Availability

Data sharing not applicable to this article as no datasets were generated or analysed during the current study.
